# Smoking during pregnancy and harm reduction in birth weight: a cross-sectional study

**DOI:** 10.1186/s12884-018-1694-4

**Published:** 2018-03-12

**Authors:** Mariana Caricati Kataoka, Ana Paula Pinho Carvalheira, Anna Paula Ferrari, Maíra Barreto Malta, Maria Antonieta de Barros Leite Carvalhaes, Cristina Maria Garcia de Lima Parada

**Affiliations:** 1 Julio de Mesquita Filho Paulista State University, Campus Universitário de Rubião Júnior, s/n CEP, Botucatu, 18618-970 Brazil; 2 Nursing Department, Botucatu Medical School Julio de Mesquita Filho Paulista State University, Botucatu, Brazil

**Keywords:** Pregnancy, Smoking, Tobacco use cessation, Birth weight

## Abstract

**Background:**

Different studies have shown the advantages of abstinence from cigarette smoking during pregnancy to promote full fetal development. Given that pregnant women do not always abstain from smoking, this study aimed to analyze the effect of different intensities of smoking on birth weight of the newborn.

**Methods:**

A cross-sectional study was adopted to explore smoking in a population of pregnant women from a medium-sized city in São Paulo state, Brazil, who gave birth between January and June of 2012. Data were collected from maternal and pediatric medical files and, where data were absent, they were collected by interview during hospitalization for delivery. For data analysis, the effect of potential confounding variables on newborn birth weight was estimated using a gamma response model. The effect of the identified confounding variables was also estimated by means of a gamma response regression model.

**Results:**

The prevalence of smoking during pregnancy was 13.4% in the study population. In full-term infants, birth weight decreased as the category of cigarette number per day increased, with a significant weight reduction as of the category 6 to 10 cigarettes per day. Compared with infants born to non smoking mothers, mean birth weight was 320 g lower in infants whose mothers smoked 6 to 10 cigarettes per day and 435 g lower in infants whose mothers smoked 11 to 40 cigarettes per day during pregnancy.

**Conclusions:**

Based on the study results and the principle of harm reduction, if a pregnant woman is unable to quit smoking, she should be encouraged to reduce consumption to less than six cigarettes per day.

## Background

Since the adoption of the Framework Convention on Tobacco by member countries of the World Health Organization in 2003, there have been important global actions to control smoking. Despite this, the smoking “epidemic” has grown in some countries because of the marketing power of the tobacco industry, population growth in countries with extensive consumption, and the number of highly dependent people who are unable to quit smoking [1].

The Centers for Disease Control and Prevention has estimated that 19.0% of American adults smoked cigarettes in 2011 [2]. The Special Survey on Smoking, a supplement to the 2008 Brazilian National Household Sample Survey, reported a smoking prevalence rate of 17.2% for people aged 15 years or older [3]. In the adult population of 27 Brazilian cities, 14.8% were smokers, and the frequency was greater for men (18.1%) than for women (12.0%) [4].

It is known that smoking can cause lung and other cancers, heart disease, stroke and many other diseases [2]. When associated with pregnancy, tobacco consumption can have even more severe effects, potentially compromising not only maternal health, but also fetal health and viability [5]. In the United States, about 20% of women are smokers at the beginning of pregnancy; however, 30.2% to 61% give up smoking in the prenatal period [6]. Women who are able to quit tend to have been light smokers [7]. There are no national Brazilian data on the prevalence of smoking during pregnancy, nor are there estimates on smoking cessation during pregnancy; however, a population-based study carried out in Santa Maria, southern Brazil, reported that 23% of pregnant women were smokers [8].

Cigarettes are among the most frequently used drugs in pregnancy [9]. A Brazilian study identified greater chance of smoking during pregnancy in women with a higher number of previous pregnancies and who did not undergo prenatal care [8].

Smoking in pregnancy is also associated with cognitive disabilities in the newborn, slower fetal growth, abortion and premature birth [8, 9].

The mechanisms through which smoking leads to negative effects during pregnancy have not been fully understood. Nicotine likely plays an important role. Nicotine causes reduction in uteroplacental circulation, leading to lower maternal weight gain and in turn, negative fetal outcomes, such as small size for gestational age, low birth weight, short stature and compromised fetal neurological development. Additionally, cigarettes and their smoke contain more than 4000 potentially toxic substances, and the combination of these toxins in cigarette smoke may be the main factor responsible for health damage [10].

Other important negative effects of smoking are seen in pregnancy and the postpartum period. During pregnancy, smoking compromises local and systemic immune responses, which in turn may be associated with adverse pregnancy outcomes [11]. Postpartum, cigarettes can cause early cessation of breastfeeding and consequences for child health and development [12].

Although there are countless studies in the literature confirming the relationship between smoking and low birth weight, they have not considered the dose–response effect of smoking on low birth weight [5, 8, 13]. In view of the high prevalence of smoking during pregnancy in Brazil, the high likelihood of adverse perinatal consequences and the difficulty of quitting, this study aimed to analyze the effect of different intensities of smoking on birth weight of the newborn.

## Methods

This cross-sectional study evaluated smoking in pregnant women from 13 small towns belonging to the “*Colegiado Pólo Cuesta*”, a health network in Botucatu, a medium-sized city (140,000 inhabitants) in southeastern São Paulo, Brazil.

In Botucatu, the Public Health Service operates 18 primary care units that provide basic health care and other health services. Childbirth care is provided by specialty obstetrics and neonatology services at a university referral hospital, which has 40 beds for pregnant/puerperal women, 24 beds for newborns, 30 beds in the Intensive Care Unit (ICU) for adults and 15 beds for neonates.

In addition to public health services, private health insurance and services are also available in Botucatu. There is one private maternity hospital with 16 beds for pregnant/puerperal women, six beds for newborns and an additional 10 beds in the ICU for both adults and neonates.

Systematic sampling was used in this study: all pregnant women admitted to give birth at either of the two maternity hospitals during the study period from January 1 to June 30, 2012, were considered eligible for the study. Only women pregnant with a single fetus were included in the study. A total of 1404 pregnant/puerperal women met those conditions. Seven women refused to participate and 84 were discharged before data collection was possible; thus, the final sample consisted of 1313 pregnant/puerperal women, representing 93.5% of the eligible study population.

All subjects gave informed written consent prior to their participation in the study, in accordance with established principles of research ethics. The study was approved by the Research Ethics Committee of Botucatu Medical School (approval number 004/2013).

The variable under investigation was smoking during pregnancy (classified as: no; yes, from 1 to 5 cigarettes per day; yes, from 6 to 10 cigarettes per day and yes, from 11 to 40 cigarettes per day. With this option, the study aimed to analyze the effect of different intensities of smoking on birth weight of the newborn compared to the birth weight of newborns from nonsmoker pregnant women. Smoking during pregnancy data were obtained from medical records (56.3%) and when they were not recorded, they were obtained during interviews (43.7%) with the puerperal women in the hospital where the birth took place. In the interviews, the question asked was: “Do/Did you smoke during gestation period? If so, how many cigarettes do/did you usually smoke per day”. For both forms of data collection, women who reported having smoked just as they did not know they were pregnant or for a short period of gestation (*n* = 6) were classified as non-smoking. Women classified as smokers during gestation were those who reported having maintained this habit throughout pregnancy.

Data were also collected on potentially confounding sociodemographic, medical and behavioral variables. Sociodemographic variables included: age (classified as ≤19 years, 20–34 years, ≥ 35 years); education (≤ 8 years, 9–11 years, ≥ 12 years); paid employment (yes/no); and presence of a partner (yes/no). Medical variables included data on obstetrical history, namely: first pregnancy, yes/no; the interval between deliveries, only for multiparous women (≤ 2 years, 3–5 years, ≥ 6 years); and pregestational overweight or obesity (based on body mass index and classified according to the Institute of Medicine) [14] (yes/no). The quality of prenatal care was also investigated using the variables: place of care (public service facility, private service facility); number of medical visits (observing that seven visits are proposed as minimum by the Brazilian Ministry of Health), (< 7 visits, 7–14 visits, ≥ 15 visits, subsequently classified into < 7 visits, ≥ 7 visits); participation in a prenatal educational group (yes/no); previous advice regarding warning signs in pregnancy (yes/no); and use of both folic acid (as of the first prenatal visit) and iron sulfate (as of the 20th week of gestation)(yes/no). Finally, the presence of any problems during gestation (yes/no) was investigated, including emotional problems; alcoholic beverage consumption; use of illegal drugs; anemia; high blood pressure, pre-eclampsia, eclampsia, or hemolysis, elevated liver enzymes, low platelet count (HELLP) syndrome; diabetes; hyperemesis; hemorrhage, bleeding, or threatened abortion; and infection, such as syphilis, urinary tract infection, toxoplasmosis, human immunodeficiency virus (HIV), or hepatitis.

Infant data were also collected to evaluate effects. The outcome variable was birth weight (g). Given the close relationship between birth weight and gestational age, the effects of smoking on term and premature newborns were studied separately [15, 16]; therefore, data were also collected on the birth condition (preterm, full-term) for stratification.

Just as for the data on smoking, all these other data were obtained from maternal or infant medical records (including prenatal care cards and records from the delivery room or the nursery) during hospital admission for delivery. Data that were not recorded were obtained by interview with the pregnant/puerperal women, also during hospital admission.

All data were collected by authorized health service professionals, under the supervision of a doctoral student in public health who was responsible for quality control. The data were input to a database and checked for consistency before statistical analysis.

The data analyses were performed in two phases. First, the effect of each possible confounding variable on newborn weight was estimated using a univariate gamma response model (crude analysis); variables with *p* < 0.20 were chosen as potential confounders for inclusion in the following multivariate analysis. In the second phase, the smoking effect, corrected for the effect of the identified confounders, was estimated using a gamma response regression model (adjusted analysis). This model was selected for its ability to simultaneously estimate the main effect and correct for the effect of potential confounders (following the asymmetric probability distribution of the outcome). Relationships were considered significant if *p* < 0.05. All analyses were performed using the Statistical Package for the Social Sciences SPSS v 20.0.

## Results

Most study participants were aged 20–34 years and had 8 to 11 years of school attendance. Considering premature and term newborns, most mothers lived with a partner respectively), employed (49.7% and 56.5%, respectively), were multiparous (57.1% and 62.0%, respectively) and prenatal follow-up had been provided by public services (75.1% and 70.4%, respectively). Among the women who had preterm delivery (*n* = 189), 59.3% had attended ≤7 medical visits; among those who delivered at term (*n* = 1124), 73.2% had attended 8–14 prenatal visits.

The prevalence of smoking was 18.0% among mothers of premature infants and 12.6% among mothers of term infants. In both groups, the median of the number of cigarettes smoked per day ranged from 1 to 40 cigarettes/day. The preterm birth rate was 14.4%. Median birth weight was 2410 g and 3250 g for premature and full-term infants, respectively (Table 1).

**Table 1:** Sociodemographic, medical and prenatal characteristics, and smoking status of pregnant women in Botucatu, Brazil

Variable	Preterm delivery (n = 189)		Term delivery (*n* = 1124)	
	N	%	N	%
Age (years)				
≤ 19	37	19.6	183	16.3
20–34	130	68.8	811	72.2
≥ 35	22	11.6	130	11.6
Education (years)				
≤ 8	54	28.6	343	30.5
9–11	118	62.4	633	56.3
≥ 12	16	8.5	147	13.1
No information	1	0.5	1	0.1
Employed				
Yes	94	49.7	635	56.5
No	93	49.2	489	43.5
No information	2	1.1	0	0.0
Live with a partner				
Yes	157	84.0	949	84.4
No	30	15.9	168	15.0
No information	2	1.1	7	0.6
First pregnancy				
Yes	81	42.9	427	38.0
No	108	57.1	697	62.0
Interval between deliveries (years)^a^				
≤ 2	28	25.9	145	20.8
3–5	34	31.5	254	36.5
≥ 6	38	35.2	272	39.0
No information	8	7.4	26	3.7
Pregestational obesity or overweight				
Yes	68	36.0	353	31.4
No	121	64.0	771	68.6
Prenatal care in public service				
Yes	142	75.1	791	70.4
No	44	23.3	333	29.6
No information	3	1.6	0	0.0
Number of prenatal medical visits				
≤ 7	86	45.5	136	12.1
8 to 14	81	42.9	923	82.1
≥ 15	4	2.1	18	1.6
No information	18	9.5	47	4.2
Alcohol consumption				
Yes	9	4.8	46	4.1
No	179	94.7	1076	95.7
No information	1	0.5	2	0.2
Illegal drug use				
Yes	5	2.6	9	0.8
No	184	97.4	1115	99.2
Participation in prenatal group				
Yes	51	27.0	240	21.4
No	126	66.7	831	73.9
No information	12	6.3	53	4.7
Advised regarding warning signs				
Yes	112	59.3	724	64.4
No	74	39.1	393	35.0
No information	3	1.6	7	0.6
Use of folic acid and iron sulfate				
Yes	104	55.0	681	60.6
No	55	29.1	443	39.4
No information	30	15.9	0	0.0
Emotional problems				
Yes	31	16.4	179	15.9
No	158	83.6	940	83.6
No information	0	0.0	5	0.5
Anemia				
Yes	5	2.6	55	4.9
No	184	97.4	1069	95.1
High blood pressure/pre-eclampsia/eclampsia/ HELLP syndrome^b^				
Yes	33	17.5	58	5.2
No	156	82.5	1066	94.8
Diabetes				
Yes	9	4.8	42	3.7
No	180	95.2	1082	96.3
Hyperemesis				
Yes	7	3.7	30	2.7
No	182	96.3	1094	97.3
Hemorrhage/bleeding/ threatened abortion				
Yes	19	10.1	49	4.4
No	170	89.9	1075	95.6
Infection				
Yes	34	18.0	222	19.8
No	155	82.0	902	80.2
Smoking in pregnancy				
No	155	82.0	982	87.4
Yes, 1 to 5 cigarettes per day	8	4.2	41	3.6
Yes, 6 to 10 cigarettes per day	10	5.3	56	5.0
Yes, 11 to 40 cigarettes per day	14	7.4	30	2.7
No information	2	1.1	15	1.3
Birth weight, median (1st and 3rd quartile) (g)	2410 (1845–2810)		3250 (2975–3565)	

^a^Total of 108 women with preterm delivery and 697 women with term delivery

^b^*HELLP* hemolysis, elevated liver enzymes, low platelet count

The relationship between potential confounders and weight of premature infants is also shown in Table 2. Attendance at ≥7 prenatal medical visits; participation in a prenatal educational group; presence of emotional problems; high blood pressure, pre-eclampsia, eclampsia or HELLP syndrome; hyperemesis; hemorrhage, bleeding or threatened abortion; and infection during pregnancy were all identified as possible confounders (*p* < 0.20).

**Table 2:** Univariate analysis of possible confounding variables influencing birth weight, in premature infants (n = 189)

Variable	β	SE	*p*	CI (β;95%)
Age at delivery (years)	5.5	18.0	0.762	(−30.4 to 41.3)
≤ 8 years of education	− 282.55	262.65	0.282	(− 797.3 to 232.24)
9 to 11 years of education	− 100.97	251.11	0.688	(− 593.14 to 391.19)
Employed	124.2	203.2	0.543	(−281.7 to 530.2)
Live with a partner	210.8	337.2	0.534	(− 462.7 to 884.4)
First pregnancy	8.0	24.7	0.745	(−41.2 to 57.3)
Interval between deliveries (years)	79.3	192.8	0.682	(− 305.8 to 464.3)
Pregestational obesity or overweight	−136.5	114.3	0.234	(−361.9 to 89.0)
Prenatal care in public service	39.0	147.9	0.793	(− 253.8 to 331.7)
≥ 7 prenatal medical visits^b^	401.7	111.7	0.000	(180.5 to 622.9)
Alcohol consumption	− 210.7	279.1	0.452	(− 763.2 to 341.7)
Illegal drug use	97.9	682.7	0.886	(− 1253.5 to 1449.2)
Participation in prenatal group^b^	203.1	118.6	0.089	(−31.7 to 437.9)
Advised regarding warning signs	−5.3	114.0	0.963	(− 230.9 to 220.3)
Use of folic acid and iron sulfate	−49.7	128.6	0.700	(− 304.3 to 204.9)
Emotional problems^b^	321.0	223.3	0.154	(− 122.5 to 764.5)
Anemia	− 465.7	449.7	0.303	(− 1358.9 to 427.5)
High blood pressure/pre-eclampsia/ eclampsia/ HELLP syndrome^a^, ^b^	− 469.5	303.7	0.126	(− 1072.7 to 133.6)
Diabetes	314.7	328.7	0.341	(− 338.2 to 967.6)
Hyperemesis^b^	− 690.8	437.4	0.118	(− 1559.5 to 177.9)
Hemorrhage/ bleeding/threatened abortion^b^	− 442.1	321.8	0.173	(− 1081.3 to 197.1)
Infection^b^	− 522.3	300.1	0.085	(− 1118.3 to 73.8)

^**a**^*HELLP* hemolysis, elevated liver enzymes, low platelet count

^**b**^Selected as potential confounder

The relationship between smoking during pregnancy and birth weight of premature infants, adjusted for potential confounders (adjusted analysis), is shown in Table 3. Again, no significant difference in birth weight was found in relation to smoking.

**Table 3:** Multivariate analysis of smoking and birth weight of premature infants (n = 189)

Variable	β	SE	*p*	CI (β; 95%)
(Intercept)	− 3791.46	587.97	0.000	(− 2639.07 to − 4943.86)
Smoking in pregnancy				
Yes, 11 to 40 cigarettes per day	− 340.03	191.32	0.076	(−715.01 to 34.94)
Yes, 6 to 10 cigarettes per day	265.27	403.49	0.511	(− 525.56 to 1056.16)
Yes, 1 to 5 cigarettes per day	− 475.24	309.48	0.125	(−1081.80 to 131.34)
No				reference
≥ 7 prenatal medical visits				
Yes	394.58	135.28	0.004	(129.44 to 659.73)
No				reference
Participation in prenatal group				
Yes	−27.69	147.23	0.851	(− 316.25 to 260.86)
No				reference
Emotional problems				
Yes	103.30	197.31	0.601	(− 283.81 to 490.41)
No				reference
High blood pressure/pre-eclampsia/ eclampsia/ HELLP syndrome^a^				
Yes	−23.82	198.03	0.904	(− 411.95 to 364.32)
No				reference
Hemorrhage/bleeding/ threatened abortion				
Yes	342.39	211.14	0.105	(−71.44 to 756.21)
No				reference
Infection				
Yes	− 312.23	186.72	0.094	(− 678.29 to 53.63)
No				reference
Gestational age (weeks)	180.68	16.32	< 0.001	(148.68 to 212.67)
Hyperemesis				
Yes	− 307.98	261.78	0.239	(− 821.06 to 205.11)
No				reference

^a^*HELLP* hemolysis, elevated liver enzymes, low platelet count

In contrast, in full-term infants the following potential confounding factors (*p* < 0.20) were identified: presence of a partner; first pregnancy; interval between deliveries; attendance at ≥7 prenatal visits; emotional problems during pregnancy; age at delivery; illegal drug use; anemia; high blood pressure, pre-eclampsia, eclampsia or HELLP syndrome; hyperemesis; and infection during pregnancy (Table 4).

**Table 4:** Univariate analyses of possible confounding variables influencing birth weight, in full-term infants (n = 1124)

Variable	β	SE	*p*	CI (β; 95%)
Age at delivery (years)^b^	−9.9	4.2	0.018	(−18.1 to −1.7)
≤ 8 years of education	35.04	47.94	0.465	(−58.92 to 128.99)
9 to 11 years of education	8.63	44.3	0.846	(−78.35 to 95.62)
Employed	24.5	45.6	0.592	(−65.2 to 114.2)
Live with a partner^b^	91.9	69.2	0.185	(−44.1 to 227.9)
First pregnancy^b^	9.1	6.0	0.132	(−2.7 to 21.0)
Interval between deliveries (years)^b^	149.8	42.9	0.001	(65.5 to 234.0)
Pregestational obesity or overweight	−55.7	478.5	0.907	(− 995.8 to 884.4)
Prenatal care in public service^b^	77.7	40.6	0.056	(−2.0 to 157.3)
≥ 7 prenatal medical visits^b^	108.4	44.7	0.016	(20.7 to 196.1)
Alcohol consumption	4.8	73.6	0.948	(− 139.6 to 149.2)
Illegal drug use^b^	− 247.6	170.7	0.147	(− 582.6 to 87.3)
Participation in prenatal group	−31.9	35.6	0.371	(− 101.8 to 38.0)
Advised regarding warning signs	−13.6	31.2	0.662	(−74.9 to 47.6)
Use of folic acid and iron sulfate	11.5	39.2	0.770	(−65.4 to 88.3)
Emotional problems^b^	81.9	54.1	0.130	(−24.3 to 188.2)
Anemia^b^	− 145.2	109.9	0.187	(−361.2 to 70.8)
High blood pressure/pre-eclampsia/eclampsia/ HELLP syndrome^a^, ^b^	− 175.5	114.5	0.126	(−400.5 to 49.6)
Diabetes	−132.3	121.2	0.275	(− 370.5 to 105.8)
Hyperemesis^b^	− 229.8	120.0	0.056	(−465.7 to 6.1)
Hemorrhage/bleeding/ threatened abortion	−105.6	113.3	0.352	(− 328.2 to 117.0)
Infection^b^	− 165.9	100.4	0.099	(− 363.1 to 31.4)

^a^*HELLP* hemolysis, elevated liver enzymes, low platelet count

^b^Selected as potential confounder

The independent effect of smoking intensity on birth weight was estimated correcting for the potential confounding variables in the adjusted regression model (Table 5). Newborn weight decreased as the category of number of cigarettes per day increased, with a significant reduction at the 6 to 10 cigarettes: when mothers smoked 6 to 10 cigarettes per day, infant weight was 320.41 g (CI 95% = − 535.51 to − 105,32) lower than that of infants born to nonsmoker mothers; when mothers smoked 10 to 40 cigarettes per day, infant weight was 435.01 g (CI 95% = − 733.16 to − 136,87) lower than that of infants born to nonsmoker mothers. When the mother smoked during pregnancy up to 5 cigarettes per day there was no effect on birth weight (*p* = 0.715).

**Table 5:** Multivariate analysis of smoking and birth weight of full-term infants (n = 1124)

Variable	β	SE	*p*	CI (β; 95%)
(Intercept)	− 750.57	927.63	0.000	(− 2568.70 to 1067.55)
Smoking in pregnancy				
Yes, 11 to 40 cigarettes per day	−435.01	152.12	0.004	(−733.16 to −136.87)
Yes, 6 to 10 cigarettes per day	−320.41	109.74	0.004	(−535.51 to − 105.32)
Yes, 1 to 5 cigarettes per day	54.81	150.09	0.715	(− 348.99 to 239.37)
No				reference
Live with a partner				
Yes	93.46	92.59	0.313	(−88.01 to 274.920
No				reference
Prenatal care in public service				
Yes	−1.10	72.10	0.988	(− 142.42 to 140.21)
No				reference
≥ 7 prenatal medical visits				
Yes	125.21	80.30	0.119	(−32.18 to 282.60)
No				reference
Illegal drug use				
Yes	− 445.41	385.79	0.248	(− 1201.55 to 310.73)
No				reference
Emotional problems				
Yes	127.67	72.15	0.077	(−13.74 to 269.08)
No				reference
Anemia				
Yes	−3.09	99.58	0.975	(−198.27 to 192.09)
No				reference
High blood pressure/pre-eclampsia/eclampsia/ HELLP syndrome^a^				
Yes	19.75	91.79	0.830	(− 160.15 to 199.66)
No				reference
Hyperemesis				
Yes	− 143.85	134.86	0.286	(− 408.18 to 120.47)
No				reference
Infection				
Yes	−53.29	70.23	0.448	(− 190.95 to 84.36)
No				reference
Age at delivery (years)	−0.77	6.14	0.900	(−12.80 to 11.25)
Gestational age (weeks)	111.48	11.23	< 0,001	(−12.80 to 11.25)
Interval between deliveries (years)	4.56	8.04	0.571	(−11.19 to 20.31)

^a^*HELLP* hemolysis, elevated liver enzymes, low platelet count

## Discussion

This study evaluated the prevalence of smoking and the relationship between birth weight and smoking intensity in a population of women who gave birth in a medium-sized city in southeastern Brazil. The impact of tabagism was evaluated using a cathegorized pattern instead of a continuous variable, because of the irregular distribution of the variable and high proportion of zeros (nonsmoker mothers). That procedure was performed so that a dilution of the smoking effect could be avoided (mean effect), and the impact of different loads of maternal smoking could be detected: 1 to 5 cigarretes per day or light smokers, 6 to 10 or medium smokers and 11 to 40 or heavier smokers.

Analysis of the premature infant data showed no statistically significant differences between the birth weight of infants born to smoking and nonsmoking pregnant women. In contrast, the analysis of full-term infants revealed a negative, dose–response effect of smoking on newborn weight. Compared with infants born to nonsmoking mothers, mean birth weight was 320 g lower in newborns whose mothers smoked 6–10 cigarettes per day and 435 g lower in newborns whose mothers smoked 11–40 cigarettes per day during pregnancy. This effect was observed even after correction for identified potential confounders, such as maternal age, presence of a partner, parity, interval between deliveries, number of prenatal medical visits, emotional problems in pregnancy, illegal drug use, anemia, high blood pressure, hyperemesis, gestational age and infection during pregnancy. Interestingly, no statistically significant differences were found in mean birth weight when mothers smoked 1–5 cigarettes per day.

An important consideration is that the accuracy of the data on smoking and the number of cigarettes smoked per day during pregnancy may limit the validity of the study findings. It is known that the number of cigarettes smoked per day can vary throughout pregnancy [17], and this was not addressed in the cross-sectional design of the present study, which relied on self-reporting at the time of delivery or medical records. Besides, women who reported having quit the habit just at the beginning of gestation were considered as nonsmokers, and the passive exposure to tobacco smoke (non investigated) was not considered, which could result in some underestimation of the smoking effect on birth weight. Nevertheless, an important negative effect was observed.

The data are representative of a single place in the southeastern region of Brazil. The prevalence of smoking in the pregnant women that was found in our study (overall prevalence of 13.4%) corroborates the importance of understanding its effects. The smoking prevalence among pregnant women in Botucatu was lower than that in non-pregnant adult women in São Paulo capital city (16.8%) and higher to the average value reported in other Brazilian capitals (12%), the only population data available for comparisons [4]. Furthermore, smoking effects are mainly a result of biological processes, and that fact also may support the generalization of our findings. Nevertheless, it is likely that in similar contexts and populations (middle-income countries with good availability of prenatal care), tobacco use during pregnancy will negatively affect term newborn weight to a similar extension as it did in the present study.

About 40% of pregnant women are estimated to quit smoking spontaneously, primarily out of concerns for fetal health but also, out of concern for their own. Others may be encouraged to quit smoking, through concerted counseling about the risks of smoking to fetus and mother that begins at the initiation of prenatal care [18]. On the whole, pregnant women are receptive to educational measures and health promotion [17] and are more likely to consider smoking cessation in the context of the frequent contact with health professionals during prenatal care [9]. Accordingly, the prenatal protocol of the Brazilian Health Ministry [16] instructs that smoking pregnant women be identified in prenatal medical visits, advised to quit and offered support to achieve this goal. As such, the findings of the study population are worrying. It is likely that not all pregnant women were appropriately counseled during their medical visits. The high prevalence of smoking in the study population shows that actions to address prevention of tobacco use in general and, particularly, during prenatal care, have been inadequate in the study region.

Despite the need for smoking cessation, it may be more challenging to achieve it during pregnancy, especially considering that a powerful psychoactive drug, nicotine, causes chemical addiction to smoking [19]. Nicotine replacement therapy has been effective in helping the addicted population to quit smoking [20] and thus, reduces harm from smoking; however, its use during pregnancy is controversial [21]. Questions remain about long-term effects and the safety of nicotine replacement therapy during pregnancy and the postpartum period [13, 21, 22].

From the perspective of practical advice for pregnant women unable to quit smoking, the study findings support the recommendation of less than six cigarettes a day to minimize the negative effects of smoking on newborn weight; however, this must be validated with further studies evaluating the effects of reduced tobacco use on birth weight and on other outcomes, such as prematurity, stillbirth and sudden infant death syndrome.

## Conclusions

The study showed that smoking during pregnancy is associated with lower birth weight in full-term infants. Smoking intensity is also important. The study found a dose–response that was significant as of the 6 to 10 cigarette-per-day category.

The high reported prevalence of smoking among women during pregnancy shows that actions to promote and support smoking cessation during pregnancy are definitely necessary in the study region. Smoke-free policies, both at a national level and globally, must remain strict, especially when related to recommendations of complete smoking cessation during pregnancy. If, however, the goal of total abstinence proves impossible, there is still an opportunity to minimize the negative effects of smoking during pregnancy on birth weight by reducing as much as possible the number of cigarettes smoked per day.

## Abbreviations

HELLP: Hemolysis, elevated liver enzymes, low platelet count
HIV: Human immunodeficiency virus
ICU: Intensive Care Unit
SPSS: Statistical package for the social sciences
